# Variations in the Life Cycle of *Anemone patens* L. (Ranunculaceae) in Wild Populations of Canada

**DOI:** 10.3390/plants5030029

**Published:** 2016-06-30

**Authors:** Vladimir Kricsfalusy

**Affiliations:** School of Environment and Sustainability, University of Saskatchewan, 117 Science Place, Saskatoon, SK S7N 5C8, Canada; vladimir.k@usask.ca; Tel.: +1-306-966-6642

**Keywords:** development, growth patterns, life cycle, *Anemone patens*, Canada

## Abstract

Based on a study of a perennial herb *Anemone patens* L. (Ranunculaceae) in a variety of natural habitats in Saskatchewan, Canada, eight life stages (seed, seedling, juvenile, immature, vegetative, generative, subsenile, and senile) are distinguished and characterized in detail. The species ontogenetic growth patterns are investigated. *A. patens* has a long life cycle that may last for several decades which leads to the formation of compact clumps. The distribution and age of clumps vary substantially in different environments with different levels of disturbance. The plant ontogeny includes the regular cycle with reproduction occurring through seeds. There is an optional subsenile vegetative disintegration at the end of the life span. The following variations in the life cycle of *A. patens* are identified: with slower development in young age, with an accelerated development, with omission of the generative stage, with retrogression to previous life stages in mature age, and with vegetative dormancy. The range of variations in the life cycle of *A. patens* may play an important role in maintaining population stability in different environmental conditions and management regimes.

## 1. Introduction

Knowledge of the life stages, also called the age states [1,2,3,4], in plant ecology can be effectively used to characterize the demography of plant populations. Age provides the timeline needed for assessing population dynamics and patterns of persistence of species. Determining the life stages has been used to provide insights on rare and common native plants [5,6,7,8,9,10], invasive species [11,12] and some plant species in relation to different management regimes [13]. Through increased understanding of the life cycles of plants, it is possible to predict the behavior of populations in particular habitats, to identify crucial stages of their ontogeny that contribute to population growth, and to target those stages as part of demographic monitoring. This approach is also relevant to conservation managers and decision makers due to its relative simplicity and fairly minimal time and resource requirements.

Another important problem of plant ecology is the role of clonality in the population dynamics of plants [14]. The benefits of clonal growth have been demonstrated to be especially important in stressful environments [15]. Therefore, knowledge of these developmental processes in the life cycle can be very useful in plant ecology when studying the role of clonal plants in vegetation communities or specific clonal plant functions [16].

**Figure 1:**
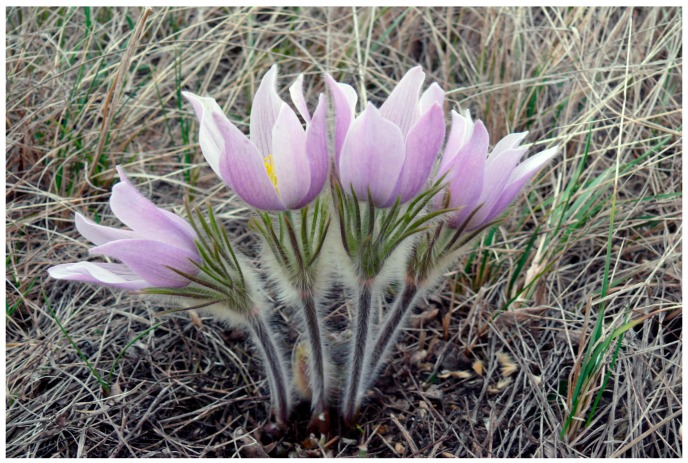
*Anemone patens* in the Northeast Swale area (Saskatoon, Saskatchewan). Photo by Vladimir Kricsfalusy.

*Anemone patens* L. (syn. *Pulsatilla patens* (L.) Mill.), the prairie crocus or pasqueflower (Figure 1), is a member of the buttercup (Ranunculaceae) family. It is a perennial herb widespread throughout the temperate regions of the Northern Hemisphere [17], covering a wide range of climatic and habitat conditions. In North America, this plant is strongly associated with native mixed-grass and short-grass prairies, which have declined to less than 20% of their original extent on the continent [18]. *A. patens* is still relatively well represented in the United States and Canada, however, its populations have declined greatly since prairie settlement [19,20]. In addition to agricultural activities, industrial and urban growth, as well as the absence of the herds of bison that once grazed on prairies and the reduction in the frequency of fires [18] substantially contributed to the species range contraction. Given increasingly growing threat to *A. patens*, its conservation status has recently been reviewed and ranked in six states of the United States and two provinces of Canada [21]. In Europe, the general population of *A. patens* is dramatically declining and hence species conservation is of special concern. It is included in the European Red List of vascular plants and in national Red Data Books of all European countries where this plant occurs [22].

Because range contraction and habitat fragmentation are threatening not only the rarest plants [23,24] studying effects of these processes on common species, like *A. patens*, is of scientific and conservation interest. There are many documented examples of previously common species that are now listed as threatened with extinction, indicating that rapid change of a common species to a rare one often occurs [24]. A good illustration of this process might be the fate of *A. patens* in Europe, where the species became threatened with extinction within just a few decades [22]. Thus, studying common native plants which are under the impact of habitat loss and fragmentation to get a better idea of how individuals and populations cope with anthropogenic impact, contributes to biodiversity science and the development of conservation management.

*A. patens* is an important plant not only from a biological and ecological perspective, but also due to its medicinal, ornamental, and cultural value, which gives another reason to study this species. *A. patens* belongs to the genus *Anemone* L., many species of which are first and foremost medicinal plants [25]. *A. patens* has been used by many native tribes in North America as a treatment for several illnesses [26,27]. A recent study reported a new triterpenoid glycoside from the roots of *A. patens*, which have long been used in traditional medicine in China [28]. *A. patens* is widely used as ornamental plants in horticulture for the decoration of rock gardens, sunny perennial borders, and cultivated beds [19]. *A. patens* is an important plant from a cultural point of view, being present in the folklore of the North American native peoples, as well as some Scandinavian and Slavic nations in Europe [27]. *A. patens* is also present in “formal” symbolism. It is adopted as the official floral emblem of the province of Manitoba in Canada, the state of South Dakota in the USA, and province of South Karelia in Finland. It also appears on the Coat of Arms of the province of Manitoba and of the City of Winnipeg [19]. The image of *A. patens* has also been profusely used in collectable items, including postage stamps in many countries and the world’s purest gold coin in Canada [29].

There is a large body of information about the biology and ecology of *A. patens* in North America. Many authors [30,31,32,33,34] conducted investigations into the pollination and phenology of *A. patens* in different regions of the United States and Canada. Several authors studied the germination and propagation of *A. patens* in greenhouse experiments [35] and in field trials on prairie remnants in Wisconsin [36] and South Dakota [37]. Intensive investigations on populations of *A. patens* have been provided in Saskatchewan. Wildeman and Steeves [38] reported the results of a detailed analysis of seasonal morphological changes and patterns of bud development in the species. Some authors studied the effect of spring fire on flower bud survival [39] and the response of *A. patens* to the time of burning [40]. Williams and Crone [41] emphasized the impact of invasive grasses on the population growth of *A. patens*. Distribution, habitat affinities and management requirements of *A. patens* were recently studied as well [20,42,43]. However, to the best of my knowledge, studies on the life history of *A. patens* in North America are lacking.

Information about the biology and ecology of *A. patens* in the Eurasian part of the species range, and impacts of different management regimes on the population growth, as well as species restoration is also quite extensive [44,45,46,47,48,49,50,51,52,53,54,55,56,57,58]. However, these publications provide only very limited data on the life cycle of *A. patens*, and they do not examine the plant development and growth patterns, its ontogenetic variations, role of clonality and seed reproduction. Also, there is a discrepancy in approaches to identification of life stages in *A. patens* by different authors [44,54,55,57]. Hence, despite many conducted studies, knowledge about the life history of *A. patens* in Eurasia remains fragmented and incomplete.

In spite of the scientific and cultural importance of *A. patens* and the rising concern over the species conservation in North America and its endangerment in most parts of Europe, assessments of the plant’s life cycle and possible causal factors of its decline are lacking. Understanding of variations in the life cycle of *A. patens* in different vegetation types or disturbances is important to work towards maintenance and recovery of its populations. Existence of distinct types of ontogenetic development in *A. patens* could be used as an indicator of its present population vitality, and also as a basis for evaluating and predicting the effects of different conservation management strategies.

Therefore, the objectives of this study on *A. patens* were to (1) analyse and describe the plant life cycle; (2) identify and characterize the life stages; (3) investigate the ontogenetic growth patterns; (4) analyse plant reproduction; and (5) identify different types of ontogeny.

## 2. Results

**Figure 2:**
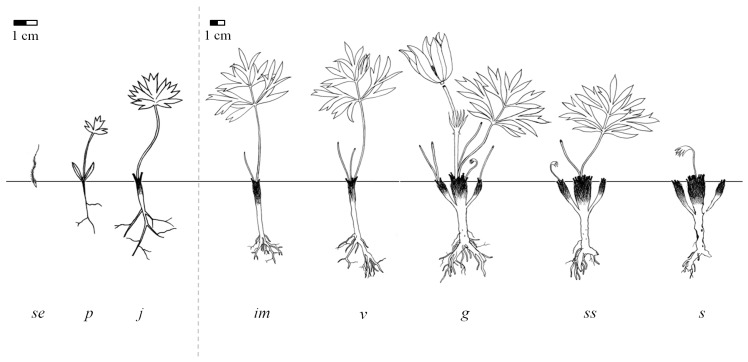
Schematic drawing of different life stages of *Anemone patens*: se—seed, p—seedling, j—juvenile, im—immature, v—virginile, g—generative, ss—subsenile, and s—senile. Drawing by Yakiv Ponomarenko.

Figure 2 shows the identified eight life stages for *A. patens*, which belong to four ontogenetic periods—latent (seed), pre-reproductive (seedling, juvenile, immature, and virginile), reproductive (generative), and post-reproductive (subsenile and senile)*.*

### 2.1. Life Stages

Seed (se). After the flower senesces and loses its sepals, the pistils develop into a cluster of achenes. Each achene is about 3 mm long, bearing a bent pilose plume; style is slender, 2–3.5 cm long. The seeds are shaped like spears and are covered with backward pointing hairs. The germination of seeds is epigeal. The cotyledon lamina is ovate (it is not shown on Figure 2). After the emergence of the cotyledons, the seed case remains in the soil. 

Seedling (p), Table 1. The plants are distinctly tap-rooted, with a thin primary root bearing few lateral roots. The cotyledons are lanceolate, the first leaf dentate or trifid, and the subsequent leaves (one or two) trifid with dentate lobes and arranged in a rosette. Rapid growth of the primary root normally begins when the cotyledons expand. The seedling stage lasts about two to three months. 

Juvenile plant (j), Table 1. The transition of a seedling to the juvenile age stage was fixed as the onset of epicotyl growth and the appearance of the first trifid true leaf. The primary shoot system consists of shortened orthotropic annual shoots that descend into the soil to a depth of 2–3 cm, and some lateral roots. The rootstock becomes thickened in its upper part and begins to accumulate dormant buds. The juvenile stage usually lasts one year; however, some plants may stay in this stage for two years or longer in unfavourable conditions, e.g., in vegetation communities with a thick litter layer.

Immature plant (im), Table 1. This age stage is characterized by a greater dissection of the leaf lamina and by further development of the root system. Immature plants have one to two leaves which are similar in shape to the leaves of mature individuals, but are less strongly dissected. There is a well-developed lateral root system. The main shoot continues to grow monopodially until the onset of sexual reproduction when its growth switches to sympodial branching. The immature stage lasts a minimum of one year. 

Virginile or vegetative plant (v), Table 2. This age stage is principally distinguished by its leaf shape, which is also characteristic of generative individuals. The leaf lamina is palmately compound. Individual vegetative plants have two to five leaves. High variability in the degree of leaf dissection and in the width of leaf segments was often observed. The vegetative stage lasts for at least three years. However, individuals may remain in this age state for many years in vegetation communities with a thick litter layer. In addition, some shoots or even whole individuals, having grown vegetatively for several years, may die without having flowered. The shoot system in young virginile plants (v_1_) undergoes a relatively rapid transition from a one-leaved to a two-leaved stage. Mature virginile plants (v_2_) always bear three leaves. Dormant buds may become active and produce lateral shoots, contributing to the branching of the rootstock. In old virginile plants (v_3_), the number of leaves increases to four or five. The mature plants have a strongly branched, somewhat woody rootstock with numerous dormant subterranean lateral buds.

Generative plant (g), Table 3. This age stage is characterized by the appearance of reproductive organs. Flowers are borne singly on the shoots and are covered in a dense indumentum. After pollination, the stalk between flower and bracts rapidly elongates, raising the developing seed head up to 11–30 cm above the ground. At the same time, the leaves fully expand.

Young generative plants (g_1_) usually produce a single flower per generative shoot. The rootstock tends to branch near its apex, forming multiple vegetative shoots. The mature generative plants (g_2_) exhibit maximal seed and vegetative productivity. The number of flowers increases to two or three per generative shoot (eight to eighteen per individual plant on average). The root system is similar to that of a young generative plant, but the upper part of the rootstock and the caudex tend to branch, with two to ten vegetative shoots present. In old generative plants (g_3_), the number of flowers decreases to one or two per generative shoot (tree to eight per individual plant on average). The rootstock is branched in its upper part, resulting in three to twelve vegetative shoots. The central part of the caudex begins to degenerate, which ultimately leads to vegetative disintegration in the subsequent (post-reproductive) life stages.

Large, well-developed individuals of *A. patens* growing in favorable conditions in cultivation begin to flower at three to four years of age. In the wild state, such plants, judging by the growth rate in the previous life stages, start flowering later, in the age of six to seven years. This study has not allowed accurate determination of how long the different generative sub–stages (g_1_, g_2_, g_3_) last. However, based on the dynamics of growth and senescence in generative individuals, *A. patens* can generally persist in the generative stage for several decades. Some generative individuals may revert to a vegetative stage after flowering and remain in it until conditions are favorable again. A limited number of my observations show that separate shoots of the same plant, as well as whole generative individuals, can go into a state of dormancy, which may last from one to five years or maybe even longer. However, the duration of this 5-year study does not allow collection of more inclusive data.

The post-reproductive (post-generative) period is the final phase in the senescence and dieback of individuals. Senescence initially causes an upsurge in flowering; over time, flowering decreases and eventually ceases altogether. Degradation of the root system during the senescent period may contribute to vegetative disintegration, but eventually the roots and rootstock decompose completely and the plant dies. This period may be divided into two distinct life stages, according to the balance of formation and degeneration of vegetative structures in generative adult individuals.

Subsenile plant (ss), Table 4. This age stage is characterized by the cessation of flowering. Occasionally, single abortive flowers may develop; these do not set seeds. The leaves, one to five, are smaller and simpler than those of generative individuals. The root system comprises a few large split rootstocks. Subsenile plants undergo vegetative disintegration; however, this rarely leads to the formation of new individuals from the separated branches. The breakdown of the central part of the caudex, at the initial branching point, begins in old generative plants. Thereafter, the process of dieback intensifies, progressing to vegetative disintegration. Disintegration leaves the individual elements (branches) of the plant interconnected only by small junctions of tissue, which then rapidly break down.

Senile plant (s), Table 4. Senile plants have ceased flowering and have a highly degraded rootstock (thus, they could only be reliably identified by excavation). Senile plants will die at the completion of the current growth period. Senile plants have, for the most part, one somewhat-pleated leaf and no flowers. The central cluster of shoots and the core of the basal part of the tap-root dies off completely.

### 2.2. Growth Patterns

**Figure 3:**
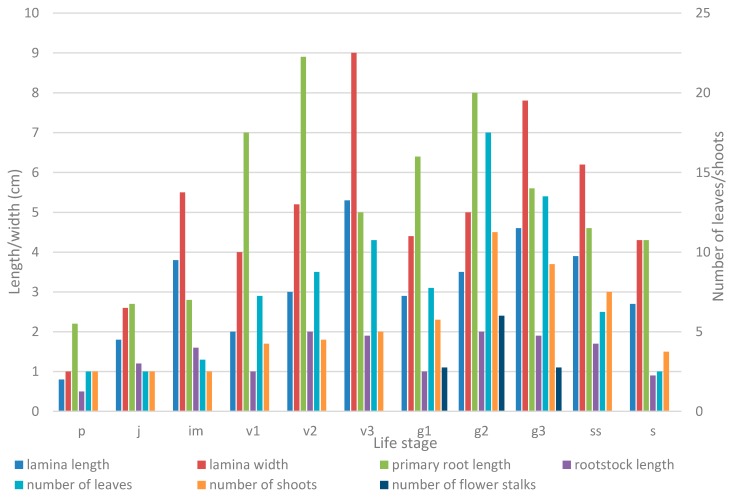
The change of some morphological characteristics during the ontogeny of *Anemone patens*. Chart represents means (*X̅*) of the study structures for each life stage: p—seedling, j—juvenile, juvenile, im—immature, v_3_—old virginile, g_3_—old generative, ss—subsenile, and s—senile. Flower stalk length (g_3_) is not shown on the figure because data are too large.

The comparison of morphological characteristics of different life stages (Table 1, Table 2, Table 3 and Table 4) during ontogeny of *A. patens* is shown in Figure 3. The maximum sizes of the morphological structures during ontogeny are most often reached in the mature generative stage (g_2_). The second peak of morphological structures is observed in the old vegetative stage (v_3_), representatives of which exceed most of the morphological features of young generative individuals (g_1_). This may be a result of the complex origin of the old vegetative stage (v_3_), which includes individuals with different ontogenetic pathways. Along with vegetative individuals that experience the complete cycle of ontogeny with the generative stage, there are also some plants with incomplete ontogeny that may grow vegetatively for several years, but never proceed to flowering. These plants are larger in size, and as such they contribute to an increase of the mean values of quantitative characteristics of the old vegetative stage. This ontogenetic stage may also include formerly generative individuals with an interruption in flowering (this can be distinguished by remnant flower stalks from the previous season). However, such plants were excluded from the analysis in order to minimize their impact on the quantitative characteristics of the old vegetative stage.

### 2.3. Reproduction

My observation on permanently marked individuals of *A. patens* shows that the senile plant experiences vegetative disintegration of the tap-root into individual ramets. This rather incomplete or partial disintegration occurs at the end of ontogeny in the post-generative phase and is not accompanied by true rejuvenation and dispersal of the offspring; thus, it does not play a substantial role in the renewal of the population. The senescing rootstock of old plants decays, causing plant disintegration into several fragments bearing parts of the main rootstock with multiple ramets that are clustered closely together. The distance between ramets increases from year to year so that each of them may eventually become an individual plant.

The rootstock disintegration in *A. patens* as described above leads to the formation of compact clumps. Although thorough rejuvenation of ramets does not occur, it is possible to distinguish three key stages in clump development: juvenility, maturity, and senescence. The daughter individuals formed during vegetative disintegration undergo incomplete rejuvenation; as a result, the clump’s senescence is delayed, and thus it may have a lifespan of several decades. 

**Figure 4:**
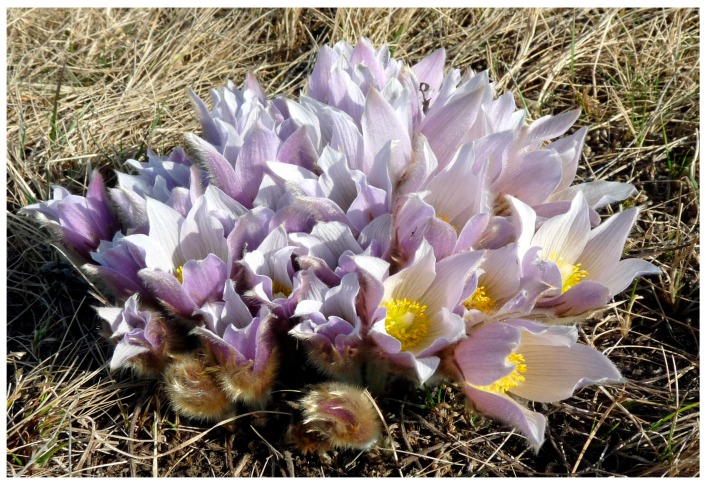
The largest clump recorded for *A. patens* growing in the Crocus Prairie Conservation Area (Saskatoon, Saskatchewan). Photo by Vladimir Kricsfalusy.

Vegetative growth in *A. patens* occurs through the complex branching patterns during July. The age of clumps varies as follows: young clumps, from two to five vegetative shoots; mature clumps, from six to ten shoots (including one to five flowering shoots); and old clumps, from ten to seventeen subsenile shoots. The largest solitary clump of *A. patens* identified in this study (Figure 4) had 111 leaves and 34 flowers (it substantially exceeded the largest known clump which formed 50 leaves and 20 flowers reported from the vicinity of Saskatoon by Wildeman and Steeves [38].

**Figure 5:**
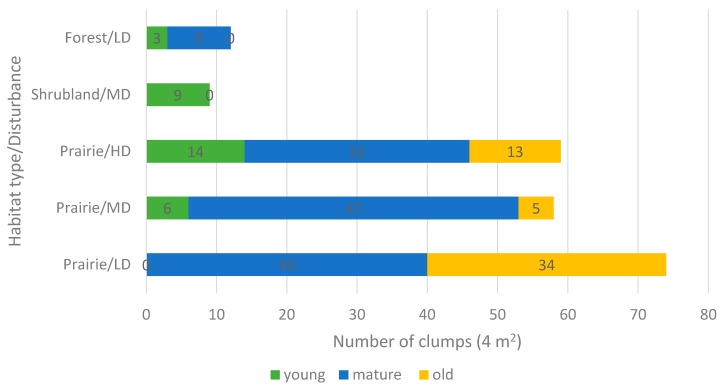
Distribution of clumps of different age in *Anemone patens* in habitats with varying levels of disturbance. Prairie: LD—low disturbance (Battlefords Provincial Park), MD—moderate disturbance (Crocus Prairie Conservation Area), HD—high disturbance (Redberry Lake Biosphere Reserve); Shrubland: M—medium disturbance (Cranberry Flats Conservation Area); Forest: LD—low disturbance (Prince Albert National Park).

Distribution of clumps of different age in the study habitats is shown in Figure 5. It seems that proper habitat conditions are critical for the growth and regeneration of *A. patens.* Our results indicate that semi-open shrubland habitats (association: *Juniperus horizontalis-Carex obtusata-Calamovilfa longifolia*) and low density forest habitats (association: *Pinus banksiana-Vaccinium myrtilloides-Cladonina arbuscular*) with limited shade and mixture of bare ground patches on light sandy soils create favourable conditions for seed reproduction. In these sites with the low to moderate level of disturbance, where sexual reproduction in the life cycle of *A. patens* is intense, there are mostly young clumps present. 

The situation is more complex for prairie habitats (associations: *Festuca hallii-Heterostipa curtiseta-Agropyron dasystachyum, Agropyron dasystachyum-Heterostipa comata-Festuca pratensis*, and *Artemisia frigida-Heterostipa comata-Koeleria macrantha-Carex* spp.). All three types of clumps were found in prairies with different levels of disturbances; however, the ratio of the clumps substantially differs (Figure 5). In sites with relatively low disturbance and little sexual reproduction of *A. patens,* there are only mature and old clumps. In moderately-grazed prairie, mature clumps prevail, while in heavily-grazed site, the number of mature clumps is comparable to the sum of young and old clumps. It seems that lack of disturbance may prevent sites for germination. Overall, the extent of clumping is substantially higher in prairie (58–74 clumps per 4 m^2^) than in shrubland and forest habitats (9–12 clumps per 4 m^2^).

The flowering period in *A. patens* lasts for about few weeks from April to May. The flowers open in sunshine and close again in the evening and in cloudy weather. The fruits are one-seeded achenes and have pappus hairs, which may enhance seed dispersal. Dispersal takes place during June over short distances (less than 30 cm from parent plants) by wind. The seeds usually germinate in fall, after the rainy summer, or next spring. Soil moisture appears to be critical for seedling establishment, as seedlings only survive in wet summers. In unsuitable conditions (i.e., during a dry summer), germination may be delayed until the following spring. 

**Figure 6:**
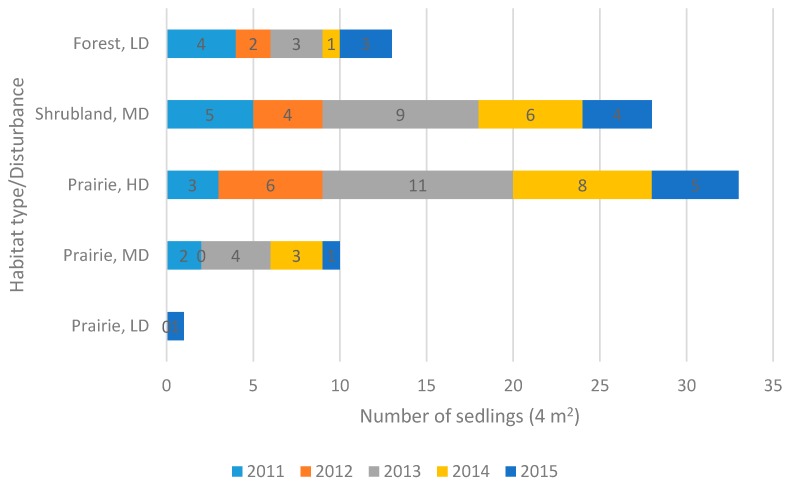
Number of seedlings of *Anemone patens* in habitats with varying levels of disturbance. Prairie: LD—low disturbance (Battlefords Provincial Park), MD—moderate disturbance (Crocus Prairie Conservation Area), HD—high disturbance (Redberry Lake Biosphere Reserve); Shrubland: MD—medium disturbance (Cranberry Flats Conservation Area); Forest: LD—low disturbance (Prince Albert National Park).

The establishment of new plants appears to be an irregular event, as seedlings (p) have been observed during this study not every year and in one population were not recorded at all (Figure 6). In shrubland and forest habitats with low cover of ground vegetation and low to moderate amount of disturbance (recreational trampling) seedling recruitment (13–28 seedlings per 4 m^2^) is more intense than in prairie habitats with low or moderate level of disturbance (1–10 seedlings per 4 m^2^).

### 2.4. Ontogeny

**Figure 7:**
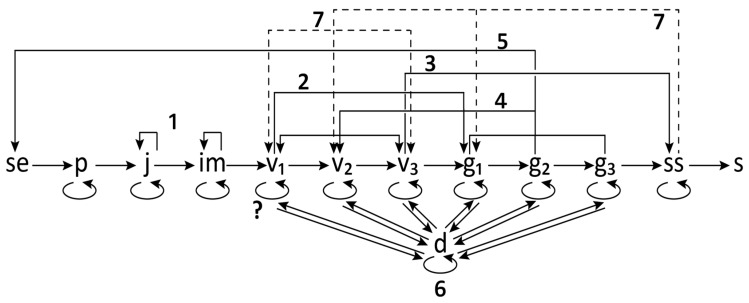
A lifecycle graph of *Anemone patens* incorporating the regular type of development and its variations: 1—with slower development in young age, 2—with an accelerated development, 3—with omission of the generative stage, 4—with retrogression to previous life stages in mature age, 5—with seed reproduction, 6—with vegetative dormancy, and 7—with optional rejuvenation in old age; life stage: se—seed, p—seedling, j—juvenile, im—immature, virginile (v_1_—young, v_2_—mature, v_3_—old), generative (g_1_—young, g_2_—mature, g_3_—old), ss—subsenile, s—senile; d—dormancy; disintegration shown by the dashed line.

The variable nature of *A. patens* development is manifested in the possibility of several variations of the ontogenetic cycle (Figure 7). Based on observations conducted, *A. patens* has slow ontogenetic growth, i.e., it takes many years for a plant to reach reproductive status. The plant undergoes a series of structural changes through its life cycle, from seed through germination, juvenility, reproductive maturity, and senescence, to death.

Within *A. patens* populations (Figure 7), besides the “standard” individuals, which undergo the regular cycle of ontogeny with seed reproduction (type 5), there are others that optional exhibit weak rejuvenation of the subsenile vegetative individuals and subsequently die (type 7). There were also observed plants with the following specific changes: with slower development, manifested in an extended juvenile and immature period (type 1); with accelerated development, which bypass certain vegetative stages and proceed to flower earlier (type 2); with only vegetative portion of the cycle and die off without flowering (type 3); with reverting from the generative to vegetative stage after flowering (type 4), and with vegetative dormancy (type 6). 

The proportion of individuals that veer away from the regular life cycle of *A. patens* was estimated in two sites (*n* = 100 for each sample) with contrasting habitat conditions—Crocus Prairie Conservation Area and Cranberry Flats Conservation Area (Table 5). It was found that the percent of individuals with different variations in the ontogeny is substantially higher in Crocus Prairie (slower development 19.2%–26.7%, development without flowering 21.3%–31.7%, development with reverting to earlier stages 12.7%–15.2%), than in Cranberry Flats (1.3%–1.9%, 0.9%–2.2%, and 1.1%–1.9% respectively). Only the proportion of individuals with accelerated rate of development was higher in Cranberry Flats (11.3%–15.7%), than in Crocus Prairie (0.1%–0.3%). Data obtained on vegetative dormancy are presented in the next paragraph. 

Dormancy in *A. patens* occurs at vegetative (v_2_, v_3_) and generative (g_1_–g_3_) developmental stages. It is still uncertain whether also the young vegetative individuals (v_1_) may become dormant. More data are needed for a more definite statement. In this survey, the percent of dormant plants varied substantially. It was much higher in Crocus Prairie (1.7%–3.2% in vegetative plants (v_2_, v_3_) and 9.7%–10.3% in generative plants), than in Cranberry Flats (0.1%–0.7% and 0.9%–1.5% respectively). Additionally, about 21% of all dormant plants in Crocus Prairie stayed in this stage over the entire period of the study. This number is substantially lower in Cranberry Flats, where only 2.7% of all dormant plants stayed in this stage for all five years. 

## 3. Discussion

Although biology and ecology of *A. patens* have been studied for many years, the fragmented knowledge about the species life cycle remained to be assembled into a coherent picture. Our study not only meets this challenge, but also reveals for the first time extensive variation in the life cycle of *A. patens*. 

There were several attempts to classify the growth form of *A. patens*. According to Wildeman and Steeves [38], *A. patens* is a long-lived hemicryptophyte with a vertical rootstock that very often branches with age. Ziman [59] classifies the species life form as semi-rosette, monopodial, caudical, tap-rooted, herbaceous, and polycarpic. Some authors [52] suggest that *A. patens* is a long-lived monoecious perennial herb with a tap root and a vertical, branched rhizome. Using classification of the types of clonal growth organs suggested by Klimešová and de Bello [60], I have identified *A. patens* as a “root-splitter”. The potential ability of *A. patens* to reproduce vegetatively by root suckers has been mentioned in the literature [47]; however, the data obtained by other authors [44,48] and my studies do not confirm this.

The plant excavation and detailed measurement of its morphological characteristics, show that young individuals of *A. patens* (p, j, im) have one to two leaves and adult plants (both vegetative and generative individuals) have three and more leaves. These findings are in line with the authors who also used plant excavation to study the species’ life cycle in the steppes of Kazakhstan [44] and in the boreal forests of Northern Europe [54]. On the other hand, Röder and Kiehl [55] and Juśkiewicz-Swaczyna [57], who studied the population structure of *A. patens* in Central Europe without excavation of individuals, suggested that young plants have one to three leaves, while the adult has more than three leaves. It should be noted that in addition to different methodological approaches, the distinctions between these study regions in climate and local geographical conditions might have been a significant factor as well.

Ontogeny in *A. patens* may last for several decades. According to Cibanova [46], plants remain vegetative until their 14th year, when the first flower shoot is produced. Based on my observations, *A. patens* has slow ontogenetic growth, but the first flowering may occur earlier, around seven years of age (Kricsfalusy, unpublished). *A. patens* has an ontogeny with a long reproductive cycle by seeds and optional subsenile vegetative disintegration. The separation of weakly rejuvenated individuals through subsenile vegetative disintegration may occur from time to time, however these individuals eventually die off and do not contribute to the renewal of populations in a significant way. Thus, vegetative reproduction by cloning does not appear to play a role in this species.

Vegetative dormancy in *A. patens*, previously not reported in the literature, is of particular interest. I consider vegetative dormancy as a time during which the plant does not sprout. According to some authors (see review by Shefferson [61]), vegetative dormancy can be seen as an extension of over-winter dormancy, in which buds form on the plant but fail to sprout. Several studies have suggested that dormancy may buffer individual plants from stress encountered above-ground [61,62]. It is likely that a probable cause of dormancy in *A. patens* is the deterioration of plant communities resulting in the build-up of a heavy layer of thatch due to lack of disturbances (grazing and fire), as was observed in prairie habitats. Semi-open shrubland habitats with limited shade and thin ground vegetation cover offer more optimal conditions for sprouting; less than optimal conditions lead to dormancy (failure to sprout). It seems that the impact of weather conditions is less important at the local level (both study sites are located less than 20 km apart). However, more experimental work needs to be done across the range of *A. patens* to identify the role and patterns of vegetative dormancy and how it occurs in different populations. 

This study shows that the distribution of clumps and their age in *A. patens* vary substantially depending on vegetation type and level of disturbance. This is adding to the evidence that *A. patens* grows very well in grazed and regularly burned prairies [40,63] and remnants of native grasslands in urban areas [42] of North America. According to Bruynooghe and Macdonald [63], *A. patens* can be an indicator of overgrazing in some prairie types. It will be interesting to broaden this study to the whole distribution range of *A. patens* in order to relate its life history traits to the large diversity of habitats occupied. Consideration of the age structure of the populations may also be very useful as it can show the response of *A. patens* against disturbances and indicate the population trend [54].

Several experimental studies on *A. patens* [45,52,53] demonstrate that this species requires a certain amount of disturbance, mostly grazing or burning, to open the layer of ground cover for successful reproduction by seeds. My observation are in line with those findings. It is likely that high density of ground vegetation and a thick layer of thatch in prairie habitats prevents seeds from reaching the soil surface and germinating by blocking sunlight and physically obstructing vertical growth. As shown in some experimental studies [40], occasional fires greatly improve growing conditions for *A. patens* by boosting the supply of nutrients and sunlight when dry grass cover is removed. The relations between seed reproduction, vegetation characteristics, soil properties and biotic factors require further detailed studies. 

The range of variations in the life cycles of *A. patens* may support the dynamic heterogeneity of populations which in turn ensures their stability in different environmental conditions and management regimes. It seems that *A. patens* may change the regular cycle of ontogeny when the habitat conditions, particularly the structure of the vegetation have become not favourable due to lack of disturbances. These findings may contribute to demographic analysis of population viability in *A. patens*, however more experimental evidence is required before any further progress can be made. Overall, given that the variation in life cycle of *A. patens* may be attributed to the structure of the vegetation cover this could have important implications for conservation and management of the species and as such requires special attention and future research.

## 4. Materials and Methods

### 4.1. Study Area

**Figure 8:**
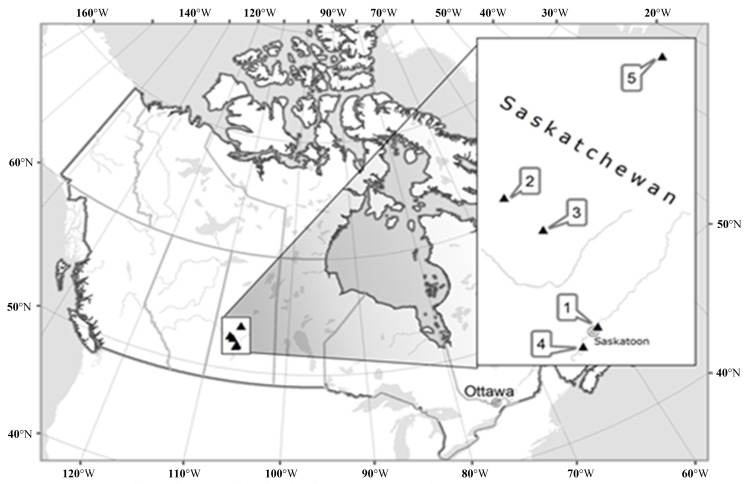
Locations of the study populations of *Anemone patens* in Saskatchewan, Canada: 1—Crocus Prairie Conservation Area, 2—Battlefords Provincial Park, 3—Redberry Lake Biosphere Reserve, 4—Cranberry Flats Conservation Area, 5—Prince Albert National Park.

Detailed field surveys were performed over the period 2011–2015 within five study sites located in Boreal Transition Ecoregion (Boreal Plain Ecozone), Mixed Grassland Ecoregion and Aspen Parkland Ecoregion (both located in Prairie Ecozone) of Saskatchewan, Canada (Figure 8, Table 5). Overall, from north to south, four ecological zones and 11 ecoregions are recognised in the province [64].

At each study site one permanent plot (10 m × 10 m) was established in different habitat types (prairie, shrubland, and forest) that harbour vegetation communities with relatively high densities of *A. patens,* in which the species cover varies from 5% to 25%.

### 4.2. Data Collection

Each of five permanent plots (Table 5) was staked out over a representative part of the *A. patens* population. For each plot the following conditions were recorded: elevation, physiognomy (aspect), slope, percent vegetation cover, litter, bare soil, and type and degree of disturbance (grazing, trampling, burning, burrowing, and presence of invasive species). Within the plot, two 4 m^2^ sampling quadrats were placed randomly in a flexible arrangement of four sub–quadrats (1 m × 1 m) each, depending on vegetation structure. To obtain measurements of different life stages and to analyze clump structure, excavation of plants within the first 4 m^2^ sampling quadrat was conducted. All individuals were mapped and carefully dug from the soil with trowels to ensure that both the aboveground portion of the plant and the entire root network were collected intact.

For each life stage and sub-stage of ontogeny from 10 to 15 plants were collected and individually placed in plastic bags for transport. In the laboratory, the following quantitative morphological characteristics were measured: (1) the number of leaves; (2) lamina length; (3) lamina width; (4) the number of shoots; (5) rootstock length; (6) primary root length; (7) the number of flower stalks per shoot. Where multiple leaves, shoots, and/or flower stalks were present, the largest of each was measured.

For each life stage and sub-stage of ontogeny from 10 to 15 plants were collected and individually placed in plastic bags for transport. In the laboratory, the following quantitative morphological characteristics were measured: (1) the number of leaves, (2) lamina length, (3) lamina width, (4) the number of shoots, (5) rootstock length, (6) primary root length, (7) the number of flower stalks per shoot. Where multiple leaves, shoots, and/or flower stalks were present, the largest of each was measured.

Investigations of the long-term ontogenetic growth patterns of *A. patens* were conducted within the second 4 m^2^ sampling quadrat. To stake out each of four sampling sub-quadrats (1 m × 1 m) at the corners metal rods were used. Within these sub-quadrats, all individuals were permanently marked with tags and mapped on graph paper at a scale of 10 mm = 1 cm. Each consecutive vegetative season, using metal finder and created graph maps, all stakes and tags from the previous year were relocated. The number of leaves and flowers on plants were counted in late June at peak of leaf mass development.

The periodization of life stages and sub-stages was carried out in correspondence with the methodological approach developed by Rabotnov [1,2] and later adopted to different plant growth forms by other authors (e.g., [3,4,7,8,13]). According to Gatsuk et al. [4], the most common features used to define the various age stages are: the type of growth, the pattern of branching of the root and shoot systems, leaf form, the presence of a particular type of shoot, the ability to reproduce by seeds, the balance between living and dead structures, the balance between actively-growing and fully-formed structures, and the manner of nutrition.

The following disturbance types suspected to affect *A. patens* were recorded for each sampling location: (1) grazing—estimated by the presence of grazed stalks or leaves and other signs of herbivore use of the area such as deer droppings and tracks; (2) burning—recent (within season) fire covering the whole population would be considered as severe while a site burned in past seasons and affecting the population marginally would be recorded as lightly disturbed; (3) recreation (trampling)—estimates the presence of trails from human use of natural areas and disturbed patches attributable to animal trampling. The levels of disturbance was assessed and scored for intensity and extent on a scale from 0 to 3 as follows: 0—absent disturbance, 1—low disturbance, 2—moderate disturbance, and 3—high/severe disturbance. This approach is based upon the data collection methodology adopted in Ecological land classification for Southern Ontario [66].

### 4.3. Data Analysis

The mean value (*X̅*) and its standard error (±*SE*) for each morphological characteristic was calculated. The coefficient of variation (*%CV*), defined as the standard deviation expressed as percentage of the mean, was used to measure the variation of the study morphological characteristics. 

## Figures and Tables

**Table 1:** Morphological characteristics of young individuals (p, j, im) of *Anemone patens* (*n* = 10 for each life stage). In the numerator is the mean (*X̅)* and its standard error (±*SE*), and in the denominator the coefficient of variation (*%СV*).

Life Stage	Parameter					
	No of Leaves	Lamina Length, cm	Lamina Width, cm	No of Vegetative Shoots	Primary Root Length, сm	Rootstock Length, cm
Seedling (p)	1 ± 0.00	0.78 ± 0.05	1.01 ± 0.08	1 ± 0.00	2.15 ± 0.25	0.54 ± 0.07
	0	22	24	0	38	43
Juvenile plant (j)	1 ± 0.00	1.82 ± 0.18	2.62 ± 0.30	1 ± 0.00	2.70 ± 0.24	1.16 ± 0.10
	0	45	52	0	28	38
Immature plant (im)	1.27 ± 0.21	3.83 ± 0.39	5.48 ± 0.49	1 ± 0.00	2.77 ± 0.37	1.63 ± 0.22
	26	33	28	0	60	30

**Table 2:** Morphological characteristics of vegetative individuals (v_1_–v_3_) of *Anemone patens* (*n* = 15 for each life sub-stage). In the numerator is the mean (*X̅*) and its standard error (*±SE*), and in the denominator the coefficient of variation (%*СV*).

Life Stage	Parameter					
	No of Leaves	Lamina Length, cm	Lamina Width, cm	No of Vegetative Shoots	Primary Root Length, сm	Rootstock Length, cm
Young vegetative plant (v_1_)	2.91 ± 0.51	4.01 ± 0.17	6.72 ± 0.37	1.7 ± 0.30	2.83 ± 0.62	1.72 ± 0.21
	11	13	17	20	42	41
Mature vegetative plant (v_2_)	3.53 ± 0.27	5.22 ± 0.19	8.89 ± 0.35	1.83 ± 0.11	3.53 ± 0.56	1.88 ± 0.30
	19	11	12	25	50	44
Old vegetative plant (v_3_)	4.30 ± 0.15	5.28 ± 0.48	8.97 ± 0.60	2.00 ± 0.26	4.97 ± 0.80	1.93 ± 0.19
	11	25	21	41	51	30

**Table 3:** Morphological characteristics of generative individuals (g_1_–g_3_) of *Anemone patens* (*n* = 15 for each life sub-stage). In the numerator is the mean (*X̅*) and its standard error (*±SE*), and in the denominator the coefficient of variation (%*СV*).

Life Stage	Parameter							
	No of Leaves	Lamina Length, cm	Lamina Width, cm	No of GenerativeShoots	Primary Root Length, сm	Rootstock Length, cm	No of Flower Stalks per Shoot	Flower Stalk Length, cm
Young generative plant (g_1_)	3.12 ± 0.31	4.38 ± 0.24	6.41 ± 0.62	2.34 ± 0.70	3.13 ± 0.61	1.78 ± 0.30	1.10 ± 0.01	20.63 ± 0.19
	34	17	30	30	62	34	29	28
Mature generative plant (g_2_)	6.89 ± 0.27	5.04 ± 0.38	7.97 ± 0.64	4.51± 0.32	6.97 ± 0.61	2.09 ± 0.26	2.40 ± 0.22	21.05 ± 1.37
	24	24	26	24	27	39	50	21
Old generative plant (g_3_)	5.44 ± 0.68	4.64 ± 0.24	7.76 ± 0.42	3.70 ± 0.30	5.55 ± 0.97	1.86 ± 0.21	1.10 ± 0.10	20.55 ± 1.86
	40	16	17	37	55	36	29	2

**Table 4:** Morphological characteristics of old individuals (ss, s) of *Anemone patens* (*n* = 10 for each life stage). In the numerator is the mean (*X̅*) and its standard error (*±SE*), and in the denominator the coefficient of variation (%*СV*).

Life Stage	Parameter					
	No of Leaves	Lamina Length, cm	Lamina Width, cm	No of Vegetative Shoots	Primary Root Length, сm	Rootstock Length, cm
Subsenile plant (ss)	2.50 ± 0.72	3.92 ± 0.97	6.15 ± 1.37	3.00 ± 0.26	4.58 ± 0.57	1.65 ± 0.45
	70	61	55	41	36	66
Senile plant (s)	1 ± 0.00	2.70 ± 0.70	4.30 ± 1.10	1.5 ± 0.34	4.30 ± 1.56	0.88 ± 0.64
	0	63	63	56	59	71

**Table 5:** Sampling locations of *Anemone patens* in Saskatchewan. Ecosites are identified based on Thorpe’s classification [65].

Population	Locality	Altitude (m a.s.l.)	Latitude/Longitude	Habitat Type	Ecosite	Disturbance Level/Type
Crocus Prairie Conservation Area	Saskatoon	496	52.16505/ −106.6042	Prairie	Mixed Grassland—Loam	Moderate/Recreation
Battlefords Provincial Park	Cochin	562	53.11370/ −108.3708	Prairie	Aspen Parkland—Loam	Low/Grazing
Redberry Lake Biosphere Reserve	Hafford	642	52.84245/ −107.6607	Prairie	Aspen Parkland—Gravelly	High/Grazing-Burning
Cranberry Flats Conservation Area	Saskatoon	495	52.03216/ −106.7062	Shrubland	Mixed Grassland—Sand dunes	Moderate/Recreation
Prince Albert National Park	Waskesiu Lake	443	53.22359/ −105.7604	Forest	Boreal Shield—Fresh sand	Low/Recreation
